# Supplementary material for: The Effects of Malonaldehyde on Quality Characteristics and Protein Oxidation of Coregonus peled (Coregonuspeled) during Storage
Source: Foods. 2023 Feb 7;12(4):716. doi: 10.3390/foods12040716 (PMC9955499; doi:10.3390/foods12040716)
Supplement: Supplementary file 1 [file foods-12-00716-s001.zip › foods-2178814-Supplementary.pdf]

Supplementary

Table S1. Changes in quality and protein oxidation in Coregonus peled during refrigeration and super chilling storage.

Days	0	1	3	5	7	9	11	13	15
**MDA (mg/kg)**									
**4°C**	**0.04±0.01^{a}**	**0.09±0.01^{b}**	**0.19±0.01^{c}**	**0.33±0.02^{d}**	**0.58±0.01^{e}**	**0.62±0.01^{f}**	**1.08±0.01^{g}**	**1.15±0.01^{h}**	**1.42±0.01^{i}**
**-3°C**	**0.03±0.011^{a}**	**0.09±0.01^{b}**	**0.12±0.02^{c}**	**0.19±0.01^{d}**	**0.28±0.01^{e}**	**0.34±0.06^{f}**	**0.54±0.01^{g}**	**0.64±0.01^{h}**	**1.11±0.03^{i}**
**T**	**ns**	**ns**	*	**	**	*	**	**	**
**pH**									
**4°C**	**6.75±0.01^{a}**	**6.68±0.02^{b}**	**6.64±0.02^{cd}**	**6.61±0.06^{c}**	**6.53±0.02^{d}**	**6.46±0.01^{e}**	**6.49±0.01^{ef}**	**6.39±0.01^{f}**	**6.28±0.05^{g}**
**-3°C**	**6.75±0.01^{a}**	**6.70±0.03^{b}**	**6.68±0.02^{c}**	**6.66±0.01^{d}**	**6.67±0.02^{e}**	**6.60±0.05^{e}**	**6.49±0.02^{e}**	**6.53±0.02^{e}**	**6.34±0.02^{f}**
**T**	**ns**	**ns**	**ns**	**ns**	**	*	**ns**	**	**ns**
**Drip loss (%)**									
**4°C**	**0.00±0.00^{a}**	**2.34±0.04^{b}**	**3.45±0.02^{c}**	**3.62±0.01^{d}**	**4.03±0.01^{e}**	**4.47±0.00^{f}**	**5.15±0.01^{g}**	**5.31±0.00^{h}**	**5.54±0.00^{i}**
**-3°C**	**0.00±0.00^{a}**	**1.62±0.00^{b}**	**2.48±0.01^{c}**	**4.44±0.00^{d}**	**5.00±0.01^{e}**	**5.33±0.00^{f}**	**7.40±0.01^{g}**	**7.45±0.00^{h}**	**8.29±0.00^{i}**
**T**	**ns**	**	**	**	**	**	**	**	**
**MFI**									
**4°C**	**22.50±2.10^{a}**	**34.80±4.80^{b}**	**47.50±5.80^{c}**	**52.00±3.10^{d}**	**62.50±3.50^{e}**	**73.60±2.10^{f}**	**83.30±1.50^{g}**	**94.10±2.10^{h}**	**95.50±3.40^{h}**
**-3°C**	**22.50±2.10^{a}**	**28.40±1.90^{b}**	**39.30±1.00^{c}**	**54.20±1.60^{d}**	**56.80±2.30^{e}**	**74.50±2.10^{f}**	**80.50±2.20^{g}**	**86.40±2.00^{h}**	**91.90±0.90i**
**T**	**ns**	**ns**	**ns**	**ns**	**ns**	**ns**	**ns**	*	**ns**
**Carbonyl (nmol/mg pro)**									
**4°C**	**1.25±0.11^{a}**	**1.38±0.05b**	**1.74±0.02c**	**1.81±0.05c**	**1.98±0.05d**	**2.20±0.04e**	**2.39±0.05f**	**2.61±0.03g**	**2.75±0.05h**
**-3°C**	**1.25±0.11^{a}**	**1.28±0.05a**	**1.67±0.03b**	**1.81±0.05c**	**1.83±0.04c**	**1.91±0.03c**	**2.01±0.04d**	**2.23±0.09e**	**2.30±0.03e**
**T**	**ns**	**ns**	*	**ns**	*	**	**	**	**
**Solubility (%)**									
**4°C**	**89.73±2.53^{a}**	**88.89±1.46^{ab}**	**85.51±2.93^{ac}**	**82.98±1.46^{cd}**	**80.44±1.46^{d}**	**79.59±2.53^{d}**	**75.37±1.46^{e}**	**73.68±3.87^{f}**	**72.84±1.46^{g}**

**-3℃**	**$89.73±2.53^{a}$**	**$86.35±2.93^{ab}$**	**$80.44±3.87^{bc}$**	**$81.28±1.46^{b}$**	**$74.53±2.53^{cd}$**	**$71.15±6.38^{d}$**	**$72.84±3.87^{d}$**	**$69.46±2.53^{e}$**	**$67.77±3.87^{f}$**
**T**	**ns**	**ns**	**ns**	**ns**	*****	**ns**	**ns**	**ns**	**ns**

Note: The different letters represent a significant difference between samples by time storage ($p < 0.05$). T: significantly different as influenced by storage temperature: *($p < 0.05$); **($p < 0.01$); ns: no significant difference ($p > 0.05$).
